# Polymethoxylated flavones from the leaves of *Vitex negundo* have fungal-promoting and antibacterial activities during the production of broad bean koji

**DOI:** 10.3389/fmicb.2024.1401436

**Published:** 2024-05-01

**Authors:** Jiayi Liu, Yetong Xu, Jianyu Yan, Liping Bai, Juan Hua, Shihong Luo

**Affiliations:** Research Center of Protection and Utilization of Plant Resources, College of Bioscience and Biotechnology, Shenyang Agricultural University, Shenyang, Liaoning, China

**Keywords:** *Vitex negundo*, broad bean paste, koji-making, microbial activity assay, polymethoxylated flavones

## Abstract

Broad bean paste is a popular condiment in Asian countries. Leaves of *Vitex negundo* Linn. were used extensively in China during the koji-making of broad bean paste. Spreading *V. negundo* leaves on raw broad beans during fermentation was able to facilitate the rapid growth of fungi to form mature koji. We isolated two strains of fungi from mature koji, and four strains of bacteria from the rotten broad beans resulting from a failed attempt. According to microbial activity assays, two polymethoxylated flavones, 5-hydroxy-3,6,7,8,3′,4′-hexamethoxy flavone (HJ-1) and 5,4′-dihydroxy-3,6,7,8,3′-pentamethoxy flavone (HJ-2) were isolated from *V. negundo* leaves, and the fungal growth promotion and inhibition of bacterial growth of these two compounds were found to improve the production of broad bean koji. This study reveals the compounds present in *V. negundo* leaves with bioactivity against important microbes in koji manufacture, and provides a theoretical basis for the application of *V. negundo* in broad bean paste production.

## 1 Introduction

Broad bean paste is a traditional condiment, and has been popular in China and other Asian countries for hundreds of years, with the annual consumption of broad bean paste in China alone exceeding 800,000 t (Yu et al., 2022; Zhao et al., 2023). The main ingredients of broad bean paste are usually broad beans (*Vicia faba* L.) and chili. The paste is formed by a natural fermentation process in the presence of various microorganisms in three main stages (Zhao et al., 2022). In the first stage, the koji is made using broad beans. The sauce fermentation stage then occurs in the presence of salt, and finally chili is added for the post-fermentation stage (Niu et al., 2018). The initial fermentation of the raw broad beans to make koji is a critical process in the production, and its success is directly related to the quality of broad bean paste. This is because koji is made by the action of the extracellular enzymatic system of the constituent fungi. The correct fungi must be present, and the conditions in the raw bean paste must be conducive to their growth (Tang et al., 2020; Ding et al., 2022; Li et al., 2023). Chemical substances able to regulate the growth of these fungi remain unidentified.

*Vitex negundo* Linn. is a small aromatic plant with five foliolate leave pattern from Verbenaceae and widely distributed around the world, as China, Japan, and South America (Zheng et al., 2012). The plant of *V. negundo* is widely used for the koji-making process of broad bean paste in China (as Hebei, Liaoning, and Sichuan province, etc.), and Sichuan have a longer history of using *V. negundo* to make koji. To make the koji, broad beans are peeled and briefly soaked, and then the beans are covered with and encased in the *V. negundo* leaves, after which they are allowed to ferment naturally. The fermentation lasts for 3–7 days, until the surface of the beans is covered with yellowish-green fungal hyphae, which marks the completion of the koji formation (Tang et al., 2020; Li et al., 2021). Using the *V. negundo* leaves in this process is believed to shorten the required fermentation time and can improve the success of the koji by controlling the microbial growth during fermentation. However, the mechanism underlying this effect and the active constituents responsible remain unclear.

The objectives of this study were to investigate potential interactions between chemical substances in *V. negundo* leaves and microorganisms during the formation of broad bean koji using chromatographic separation, nuclear magnetic resonance (NMR) spectroscopy analyses, strain isolation, and bioactivity assay. We identified two polymethoxylated flavones from *V. negundo* which were able to promote fungal growth and inhibit bacterial growth, and which improve the success of the formation of broad bean koji.

## 2 Materials and methods

### 2.1 Materials and chemicals

*Vitex negundo* Linn. and *Vicia faba* L. (broad bean) were used in this study. *V. negundo* plants were collected from the areas close to Fuxin City and Sichuan City, China, in August 2021, and were identified by Professor Bo Qu. Voucher specimens of *V. negundo* were made and are stored at the College of Bioscience and Biotechnology, Shenyang Agricultural University, under the numbers SYNUB015940–SYNUB015942. Raw broad beans were purchased from Chengdu (Sichuan, China) in September 2021. All analytical reagents were purchased from Sinopharm Chemical Reagent Co., Ltd (Shanghai, China).

### 2.2 Production of broad bean koji and the effect of *V. negundo* on the production process

Raw broad beans (100 g) were peeled and soaked in 200 mL clean water for 30 min. The wet beans were then placed in a sterile culture dish lined with 200 g fresh *V. negundo* leaves on the bottom. A further 200 g of fresh *V. negundo* leaves were used to cover these broad beans and to completely encase them. After that, the beans were incubated at 25°C. Any visible changes in the broad beans were noted, and the time taken for the koji to form (the appearance of yellowish-green fungal hyphae on the beans surface) was recorded. A moistened sterile gauze was applied to the broad beans instead of the *V. negundo* leaves but used in the same way in the koji-making process as a control. All experiments were conducted in triplicate.

### 2.3 Isolation and identification of fungi responsible for broad bean koji

In order to isolate the fungal strains important for the formation of broad bean koji, potato dextrose agar (PDA) medium was used to isolate the dominant fungal strains in the koji. The broad bean koji with the best fermentation state was selected, and its surface mycelium was scraped using a sterile loop and inoculated onto PDA medium. We used both molecular biological methods and morphological observation to purify the dominant fungal strains in broad bean koji following a previously described method (Jia et al., 2022). The purified strains were cultivated in PDA medium at 28°C for 72 h to allow the mycelium to grow, and the mycelium was then collected and ground in liquid nitrogen. Genomic DNA was then extracted from the mycelium using an Ezup Column Fungi Genomic DNA Purification Kit (Sangon Co., Shanghai, China). The primers ITS1 and ITS4 were used to amplify the ITS region of each of the isolated fungal strains. The obtained PCR fragments were sequenced by Sangon (Shanghai, China) and were compared with those of known species available in the NCBI database. A neighbor-joining phylogenetic tree was constructed using the generated ITS sequences in MEGA version 7.0.

### 2.4 Isolation and identification of bacteria in the rotten broad beans resulting from failure of the koji

The bacteria in the broad beans that had rotted as a result of failure of the koji-making process were isolated according to a previous method with some modifications (Niu et al., 2018). Briefly, the 1 g sample of rotten broad bean was used and mixed well with 10 mL sterile distilled water to acquire a bacterial suspension. The suspension was then diluted for 10^5^ times. Then, 0.2 mL of this dilution was spread over solid Luria-Bertani (LB) medium supplemented with 50 μg/ml of nystatin. After cultivation at 37°C for 24–72 h, colonies were isolated and purified according to their morphological characteristics. The genomic DNA of the isolated bacterial strains was extracted and the 16s rDNA was amplified using PCR with the universal primers 27F and 1492R. The PCR products were sequenced (Sangon, Shanghai, China) and identified using BLAST searches of the NCBI database. Finally, a neighbor-joining phylogenetic tree was constructed in MEGA version 7.0.

### 2.5 Petroleum ether and methanol extraction of chemicals from *V. negundo* leaves

A total of 5 kg fresh *V. negundo* leaves were subjected to a cold petroleum ether immersion extraction. The extraction was carried out over 24 h and was repeated three times. The resulting solution was evaporated to dryness at 40°C in a rotary evaporator. Then these leaves were dried in a cool place and subjected three times to methanol extraction. The resulting solution was evaporated to dryness at 50°C in a rotary evaporator.

### 2.6 The effect of *V. negundo* extracts on bacteria and fungi

Six strains of bacteria were chosen to test the biological activity of the petroleum ether and methanol extractions. The bacterial strains included *Staphylococcus aureus*, *Bacillus subtilis*, *B. wiedmannii* cdxj-1, *Pseudomonas putida* cdxj-2, *B. albus* cdxj-3, and *B. aerius* cdxj-4. The *S. aureus* and *B. subtilis* were acquired from the Agricultural Culture Collection of China. Four strains, *B. wiedmannii* cdxj-1, *P. putida* cdxj-2, *B. albus* cdxj-3, and *B. aerius* cdxj-4, were isolated from the rotten broad beans from the failed koji. The petroleum ether and methanol extracts of *V. negundo* leaves were each dissolved in methanol to a maximum concentration of 0.5% (v/v). The extracts were tested based on a previously described method (Jia et al., 2022). The 96 well plates were placed in 37°C thermostatic culture, and the absorbance at OD_625_ was recorded with a spectrophotometer every twelve hours. Finally, the growth promotion/inhibition ratio (%) was calculated according to the formula [OD_625_ (negative control)–OD_625_ (test group)| /OD_625_ (negative control)] * 100. If inhibitory activity was indicated, the half-maximal inhibitory concentration (IC_50_) was analyzed using SPSS 22.0.

Two strains of fungi were used to conduct the activity bioassay of the extractions. The fungi chosen were *Rhizopus* sp. CD-1 and *Aspergillus* sp. CD-3, both of which were derived from high-quality broad bean koji. The experiment was carried out as previously described with some slight modifications (Yang et al., 2022). The extract was dissolved in methanol and added to a 96-well plate, and the final chemical concentration set to 512–16 μg/mL through twofold serial dilutions. Pure methanol was used as a control. The absorbance at 600 nm (OD_600_) of All cultures was measured every 12 h.

### 2.7 Isolation and identification of chemical substances in the *V. negundo* petroleum ether extract

The petroleum ether extract (120 g) was passed through a silica gel column and eluted using various solutions with different polarity (dichloromethane/acetone: 1:0, 9:1, 4:1, and 1:1). Four fractions were obtained: Fr.1–Fr.4. Based on its biological activity, Fr.3 (40 g) was selected for further investigation. Fr.3 was subjected to chromatography on an MCI gel column with methanol/water (6:4, 7:3, 8:2, 9:1, and 10:0) gradient elution. Five fractions (Fr.3-1–Fr.3-5) were obtained and tested for their biological activity. Based on the results of the biological activity assays, Fr.3-1 was selected for further investigation. A total of 18 g Fr.3-1 was subjected to repeated chromatography on a silica gel column and a sephadex LH-20 column, and was finally purified using semi-preparative HPLC (Agilent 1260; X-bridge BEH C_18_ OBDTM Prep Column 130A, 5 μm, 10 mm × 250 mm; methanol in water; 3 mL/min) to obtain two compounds, named compound HJ-1 (260 mg) and compound HJ-2 (220 mg). The chemical structure of these two compounds was determined using nuclear magnetic resonance (NMR). Spectroscopic analysis of purified compounds was described as follow.

Spectra data of 5-hydroxy-3,6,7,8,3′,4′-hexamethoxy flavone (HJ-1) yellow crystals, ^1^H NMR (600 MHz, methanol-*d*_4_) δ: 7.88 (dd, *J* = 8.5, 1.9 Hz, 1H, H-6′), 7.81 (d, *J* = 1.9 Hz, 1H, H-2′), 7.14 (d, *J* = 8.5 Hz, 1H, H-5′), 4.06 (s, 3H, 7-OCH_3_), 3.93 (s, 3H, 4′-OCH_3_), 3.92 (s, 3H, 3′-OCH_3_), 3.89 (s, 3H, 6-OCH_3_), 3.88 (s, 3H, 8-OCH_3_), 3.85 (s, 3H, 3-OCH_3_). ^13^C NMR (150 MHz, methanol-*d*_4_) δ: 179.5 (C-4), 156.0 (C-2), 154.9 (C-7), 153.1 (C-4′), 150.4 (C-3′), 147.5 (C-5), 144.6 (C-9), 139.9 (C-3), 128.5 (C-6), 128.2 (C-8), 124.5 (C-1′), 123.5 (C-6′), 112.6 (C-5′), 112.4 (C-2′), 111.5 (C-10), 62.4 (6-OCH_3_), 61.9 (7-OCH_3_), 60.9 (8-OCH_3_), 60.6 (3-OCH_3_), 56.5 (2C, 3′, 4′-OCH_3_).

Spectra data of 5,4′-dihydroxy-3,6,7,8,3′-pentamethoxy flavone (HJ-2) yellow crystals, ^1^H NMR (600 MHz, chloroform-*d*) δ: 12.39 (s, 1H, 5-OH), 7.79 (dd, *J* = 10.4, 1.9 Hz, 1H, H-6′), 7.26 (s, 1H, H-2′), 7.07 (d, *J* = 8.3 Hz, 1H, H-5′), 6.03 (s, 1H, 4′-OH), 4.11 (s, 3H, 7-OCH_3_), 3.99 (s, 3H, 3′OCH_3_), 3.96 (s, 3H, 6-OCH_3_), 3.95 (s, 3H, 8-OCH_3_), 3.88 (s, 3H, 3-OCH_3_). ^13^C NMR (150 MHz, chloroform-*d*) δ: 179.2 (C-4), 155.9 (C-2), 152.9 (C-7), 149.1 (C-5), 148.5 (C-4′), 146.4 (C-3′), 144.8 (C-9), 138.6 (C-3), 136.1 (C-6), 132.8 (C-8), 122.9 (C-6′), 122.6 (C-1′), 114.7 (C-5′), 110.8 (C-2′), 107.4 (C-10), 62.1 (6-OCH_3_), 61.7 (7-OCH_3_), 61.2 (8-OCH_3_), 60.1 (3-OCH_3_), 56.0 (3′-OCH_3_).

### 2.8 The biological activities of compounds HJ-1 and HJ-2 isolated from *V. negundo* toward bacteria and fungi

Assays of biological activity toward several strains of bacteria and fungi were conducted using the purified compounds HJ-1 and HJ-2. Six strains of bacteria (*Staphylococcus aureus*, *Bacillus subtilis*, *B. wiedmannii* cdxj-1, *P. putida* cdxj-2, *B. albus* cdxj-3, and *B. aerius* cdxj-4) and two strains of fungi (*Rhizopus* sp. CD-1 and *Aspergillus* sp. CD-3) were tested. The effects of the two compounds were analyzed following the method described above.

### 2.9 Quantification of the compounds HJ-1 and HJ-2 in fresh *V. negundo* leaves

A 0.5 g sample of fresh *V. negundo* leaves was subjected to extraction for 45 min in an ultrasonic bath in the presence of 4 mL methanol. The extraction was repeated twice. The samples were centrifuged at 8,000 rpm for 10 min, and the supernatants were collected and evaporated to dryness. The extractions were re-dissolved in 1 mL methanol and used for HPLC (Agilent 1260) analysis. All experiments were repeated three times. HPLC working conditions were as follows: 10 μL extraction was added to an Eclipse XDB-C_18_ column (5 μm, 4.6 × 250 mm). The column temperature was maintained at 35°C and the compounds were monitored at 210 nm and 254 nm. The flow rate was maintained at 1 mL/min, and (A) water and (B) methanol were used as the mobile phase (0–30 min: linear 5–95% of B, 30–40 min, isocratic 95% of B).

The pure compounds HJ-1 and HJ-2 were dissolved in methanol, and a series of solutions of different concentrations (1,000, 600, 400, 200, 100, and 50 μg/mL) was generated. Standard curves were established from the HPLC analysis. The calibration curve equations for HJ-1 and HJ-2 were *y* = 0.0767*x*–28.5981 (*t*_*R*_, 29.28 min; *R*^2^ = 0.997), *y* = 0.0358*x*–6.0384 (*t*_*R*_, 27.70 min; *R*^2^ = 0.997), respectively.

### 2.10 Statistical analysis

All data were analyzed using SPSS 22.0 and graphics were created in GraphPad Prism. If the data followed a normal distribution, an independent-samples *t*-test was used for comparison between the two groups. Differences were considered to be statistically significant at *P* < 0.05.

## 3 Results

### 3.1 The use of *V. negundo* leaves promotes the formation of broad bean koji

**FIGURE 1:**
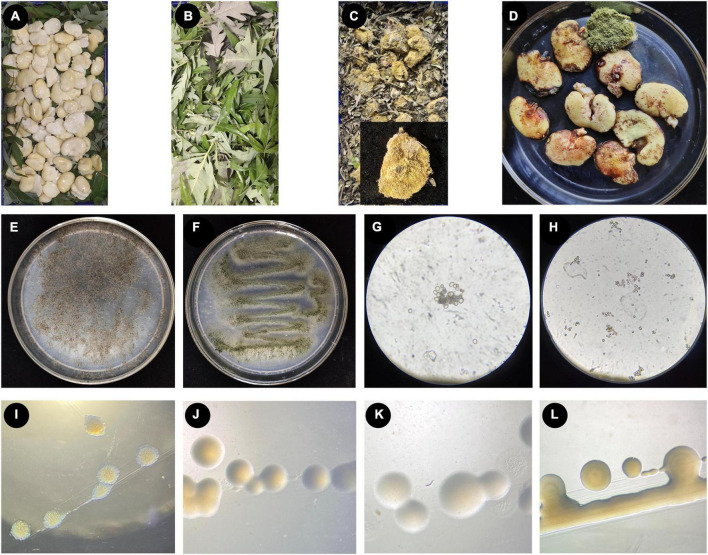
The process of making broad bean koji, and the microorganisms isolated from broad bean koji. **(A–D)** The koji-making process: raw soaked and peeled broad beans were laid in a sterile culture dish **(A)**, the beans were completely covered with *V. negundo* leaves **(B)**, example of a mature broad bean koji from the *V. negundo* treatment group **(C)**, the mature koji from the control group treated with a moistened sterile gauze **(D)**. **(E–H)** Two strains of fungi isolated from broad bean koji made using *V. negundo* leaves, including the colony morphology of *Rhizopus* sp. CD-1 **(E)** and *Aspergillus* sp. CD-3 **(F)** and the germinating spores of *Rhizopus* sp. CD-1 **(G)** and *Aspergillus* sp. CD-3 **(H)**. **(I–L)** Four strains of bacteria isolated from the rotten broad beans from failed koji, including *B. wiedmannii* cdxj-1 **(I)**, *P. putida* cdxj-2 **(J)**, *B. albus* cdxj-3 **(K)**, and *B. aerius* cdxj-4 **(L)**.

To investigate the effect of *V. negundo* leaves on the formation of broad bean koji, visible changes in the broad beans were recorded and the time taken for the koji to form was measured (Figures 1A–D). The control groups, which were treated with a moistened gauze, took an average of seven days for the koji to form. The group treated with *V. negundo* leaves formed koji in an average of five days, which was obviously shorter than the control group. The formation rate of broad bean koji was superior to control after using *V. negundo* leaves (Figures 1C, D). Thus, we found that *V. negundo* leaves significantly promoted the formation of broad bean koji.

### 3.2 Isolation and identification of the dominant fungi in broad bean koji

Two strains of fungi (CD-1 and CD-3) were acquired from mature broad bean koji, and the colony morphology of these two strains is shown (Figures 1E–H). According to morphological observations (Figures 1E, G) and sequence alignment, the fungus CD-1 was identified as belonging to *Rhizopus* sp. (sequence similarity 99%) and was named *Rhizopus* sp. CD-1. The phylogenetic placement of *Rhizopus* sp. CD-1 was established based on 15 fungal DNA sequences from the NCBI database (Supplementary Figure 1). ITS sequence analysis suggested that the strain CD-3 shared 98% similarity with *Aspergillus* sp. (Figures 1F, H), and CD-3 was named *Aspergillus* sp. CD-3. The phylogenetic placement of *Aspergillus* sp. CD-3 was established based on 15 fungal DNA sequences from the NCBI database (Supplementary Figure 2).

### 3.3 Isolation and identification of the dominant bacteria in the rotten broad beans resulting from failed koji

Rotten beans were selected for isolation of the bacteria responsible for spoilage in failed koji. Four strains of bacteria (cdxj-1, cdxj-2, cdxj-3, and cdxj-4) were obtained from the rotten beans (Figures 1I–L). From comparison with the sequences in the NCBI database, we found that cdxj-1 shared 99.93% sequence similarity with *Bacillus wiedmannii*, and it was named *B. wiedmannii* cdxj-1 (Supplementary Figure 3). The sequence of cdxj-2 shared 100% similarity with *Pseudomonas putida*, and was therefore named *P. putida* cdxj-2 (Supplementary Figure 4). The strains cdxj-3 and cdxj-4 shared sequence similarities of 99.93 and 99.79% with *B. albus* and *B. aerinus*, respectively, and were named *B. albus* cdxj-3 and *B. aerius* cdxj-4 (Supplementary Figures 5, 6).

### 3.4 Petroleum ether and methanol extractions of *V. negundo* leaves inhibit bacterial growth

The biological activities of the petroleum ether and methanol extractions were tested on six strains of bacteria.

**FIGURE 2:**
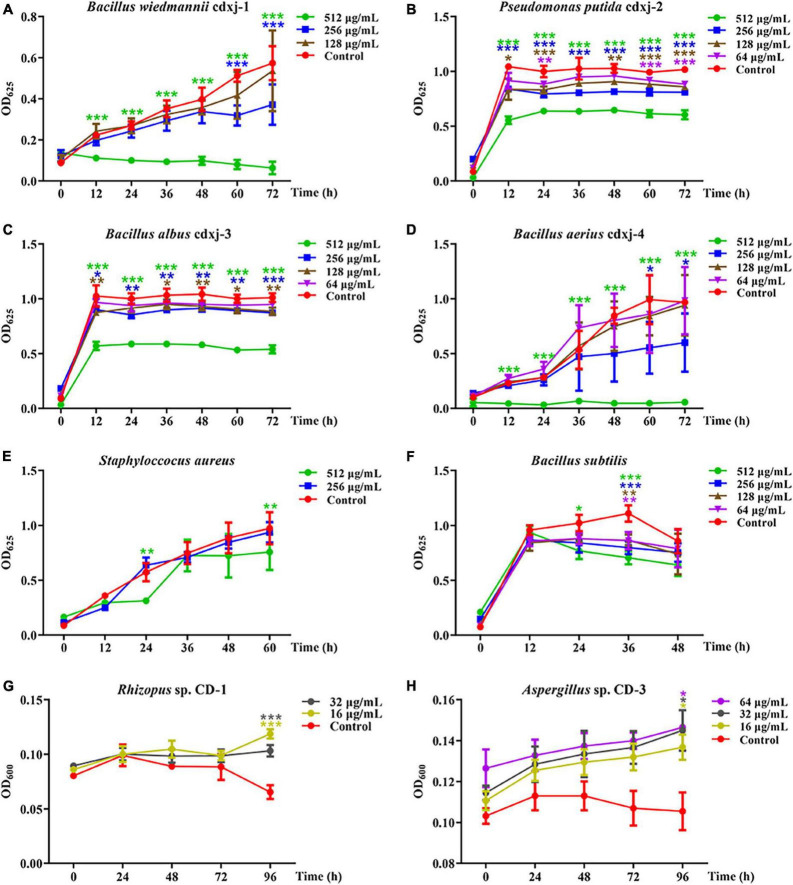
The biological activities of the petroleum ether extract of *Vitex negundo* leaves toward different strains of bacteria and fungi. **(A–F)** The petroleum ether extract of *V. negundo* leaves inhibits the growth of several strains of bacteria, including *B. wiedmannii* cdxj-1 **(A)**, *P. putida* cdxj-2 **(B)**, *B. albus* cdxj-3 **(C)**, *B. aerius* cdxj-4 **(D)**, *S. aureus* **(E)**, and *B. subtilis* **(F)**. **(G,H)** The petroleum ether extract of *V. negundo* leaves promotes the growth of certain species of fungi, including *Rhizopus* sp. CD-1 **(G)** and *Aspergillus* sp. CD-3 **(H)**. Triple asterisks indicate significant differences between the control group and other treatments, ***p < 0.001, **p < 0.05, *p < 0.01.

The petroleum ether extraction (PEE) showed various degrees of inhibition activity against the six test strains of bacteria. The growth of *B. wiedmannii* cdxj-1 was significantly inhibited after co-culture with 256 μg/mL PEE at 48 h (Figure 2A). Inhibition of *B. wiedmannii* cdxj-1 reached a peak at 72 h, with an inhibition rate of 88.95 ± 5.27%, and an IC_50_ value of 255.52 ± 58.12 μg/mL. *P. putida* cdxj-2 was only moderately inhibited by PEE, with a maximum inhibition rate of 40.60 ± 4.01% following 72 h treatment with concentrations of 512 μg/mL and an IC_50_ value of 1,213.92 ± 271.75 μg/mL (Figure 2B). A slight inhibitory effect of 46.59 ± 3.72% was observed in *B. albus* cdxj-3 following 72 h treatment with PEE at 512 μg/mL, with IC_50_ value of 2,331.89 ± 1,339.23 μg/mL (Figure 2C). Following 36 h of co-culture with PEE at 256 μg/mL, PEE exhibited evident inhibitory activity against *B. aerius* cdxj-4, with the inhibition rate reaching 90.13 ± 2.52% under PEE concentrations of 512 μg/mL at 72 h. The IC_50_ value was 184.31 ± 66.86 μg/mL (Figure 2D). *S. aureus* was found to be only slightly inhibited by PEE at the concentration of 512 μg/mL, with an inhibition rate of 45.38 ± 3.85% at 24 h (Figure 2E). PEE had a weak inhibitory effect against *B. subtilis*, with an inhibition rate of 36.32 ± 5.38% at 512 μg/mL, at 48 h (Figure 2F), and with the inhibitory effect gradually weakening as the test concentration decreased.

The methanol extract (ME) of *V. negundo* leaves demonstrated inhibitory activity against *B. wiedmannii* cdxj-1, with an inhibition rate of 71.03 ± 8.09% at 512 μg/mL after 72 h (Supplementary Figure 7A). In contrast, the growth of *P. putida* cdxj-2 and *B. albus* cdxj-3 co-cultured with ME did not differ from that of the control (Supplementary Figures 7B, C). *B. aerius* cdxj-4 was sensitive to ME, and its growth was visibly inhibited by 256 μg/mL ME at 36 h (Supplementary Figure 7D). After 72 h of co-culture with ME, *Bacillus* sp. cdxj-4 inhibition rates reached 31.32 ± 10.55% and 87.24 ± 11.67% at 256 μg/mL and 512 μg/mL, respectively. ME weakly inhibited the growth of *S. aureus* at a concentration of 512 μg/mL at 60 h, with an inhibition rate of 66.33 ± 1.99% (Supplementary Figure 7E). ME did not have an effect on the growth of *B. subtilis* (Supplementary Figure 7F).

These results suggested that certain chemical substances in *V. negundo* have significant antibacterial activities, and are particularly effective against the bacteria from the rotten broad beans responsible for failed koji. These bioactive chemicals were more effectively extracted by petroleum ether than by methanol.

### 3.5 The petroleum ether extract of *V. negundo* leaves shows growth-promoting activity toward certain fungal strains

Because the extraction of *V. negundo* compounds with petroleum ether (PEE) had a greater effect against six strains of bacteria than the methanol extraction, we decided to continue our experiments with the PEE. PEE was tested on the fungi *Rhizopus* sp. CD-1 and *Aspergillus* sp. CD-3 isolated from high quality broad bean koji. The germination of *Rhizopus* sp. CD-1 spores was promoted by co-culture for 72 h with PEE (Figure 2G), and when cultured for 96 h, the promotion rates were 77.13 ± 14.29% and 58.93 ± 24.09% at PEE concentrations of 16 and 32 μg/mL, respectively. PEE was also able to promote the germination of *Aspergillus* sp. CD-3 (Figure 2H). Three different concentrations (16, 32, and 64 μg/mL) were able to promote the growth of this fungus, with promotion rates of 29.70 ± 5.89%, 30.96 ± 12.99%, and 29.86 ± 8.24% at 96 h, respectively. Hence, PEE can promote the germination of both *Rhizopus* sp. CD-1 and *Aspergillus* sp. CD-3 spores, with the effect on the germination of *Rhizopus* sp. CD-1 being stronger than on that of *Aspergillus* sp. CD-3. These results demonstrate that the chemical components in the PEE played a role in inhibition of harmful bacteria and promotion of the germination of necessary fungal spores during the production of broad bean koji.

### 3.6 Fraction 3 from the *V. negundo* petroleum ether extraction demonstrates bacterial inhibitory and fungal promotional activities

The petroleum ether extract of *V. negundo* leaves exhibited better activity toward the target microorganisms than the methanol extract, and we therefore used the PEE in further experiments. The PEE was initially separated on a silica gel column to obtain four fractions, Fr.1–Fr.4. Fr.1–Fr.4 were used in microbial activity assays to assess their performance toward six strains of bacteria and two strains of fungi. Fr.3 was found to inhibit bacterial growth and to promote fungal growth.

**FIGURE 3:**
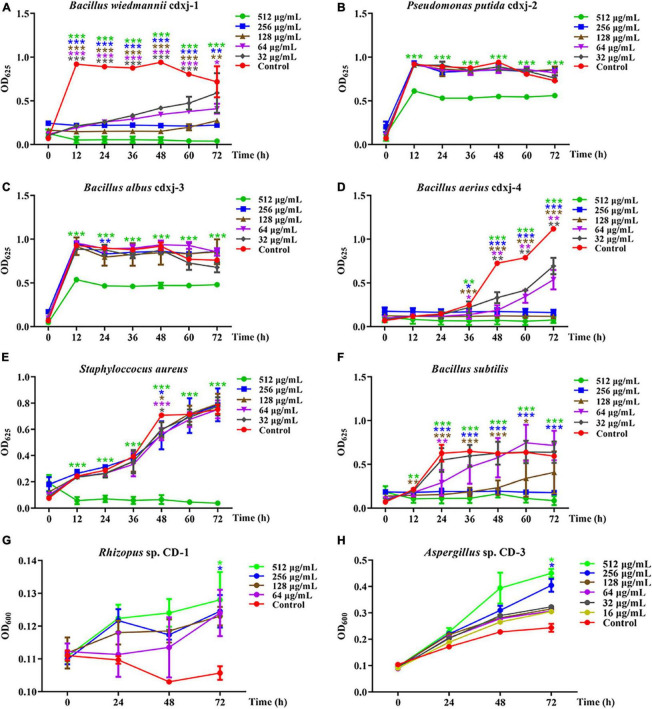
The biological activity of extract Fr.3 from *Vitex negundo* leaves toward bacteria and fungi. **(A–F)** Fr.3 inhibits the growth of several bacterium strains, including *B. wiedmannii* cdxj-1 **(A)**, *P. putida* cdxj-2 **(B)**, *B. albus* cdxj-3 **(C)**, *B. aerius* cdxj-4 **(D)**, *S. aureus* **(E)**, and *B. subtilis* **(F)**. **(G,H)** Fr.3 promotes the growth of the fungi *Rhizopus* sp. CD-1 **(G)** and *Aspergillus* sp. CD-3 **(H)**. Triple asterisks indicate significant differences between the control group and other treatments, ***p < 0.001, **p < 0.05, *p < 0.01.

Within 60 h of co-cultivation, Fr.3 showed significant inhibitory activity against *B. wiedmannii* cdxj-1 at a concentration of 32 μg/mL, and the inhibitory effect was gradually enhanced with increasing concentrations of Fr.3 (Figure 3A), with the inhibition rate at 72 h reaching 94.54 ± 2.78% at a concentration of 512 μg/mL. The IC_50_ value was 86.46 ± 15.74 μg/mL. *P. putida* cdxj-2 was weakly inhibited by Fr.3 (Figure 3B), and 512 μg/mL Fr.3 only resulted in an inhibition rate of 24.42 ± 3.16% at 72 h. Similarly, Fr.3 also showed a certain degree of inhibitory activity toward the growth of *B. albus* cdxj-3 (Figure 3C), with an inhibition rate of 36.71 ± 2.66% at 72 h and at 512 μg/mL. The antibacterial activity of Fr.3 against *Bacillus* sp. cdxj-4 was significant (Figure 3D). Fr.3 began to show inhibitory activity against *B. aerius* cdxj-4 after co-cultivation for 36 h, at 32 μg/mL. At 72 h, the most significant inhibitory effects appeared with an IC_50_ value of 45.84 ± 9.50 μg/mL, and the inhibitory rates ranged from 38.08 ± 8.43% to 93.29 ± 3.00% at 32–512 μg/mL. The growth of *S. aureus* was also obviously inhibited by Fr.3 at a concentration of 512 μg/mL and at 72 h, with an inhibition rate of 95.03 ± 1.97% (Figure 3E). The antibacterial activity of Fr.3 against *B. subtilis* was clear, and delayed the logarithmic growth phase of *B. subtilis* at 64 μg/mL (Figure 3F). Within 48 h, *B. subtilis* growth was almost halted at a Fr.3 concentration of 128 μg/mL, and at a concentration of 512 μg/mL, Fr.3 exhibited an inhibitory rate of 86.03 ± 8.47% and an IC_50_ value of 115.08 ± 87.08 μg/mL at 72 h.

Furthermore, Fr.3 promoted the germination of *Rhizopus* sp. CD-1 spores at the tested concentration range of 16–512 μg/mL, with the promotional effect being gradually enhanced with increases in concentration and time (Figure 3G). At 72 h, Fr.3 promotion rates of *Rhizopus* sp. CD-1 spore germination were 13.25 ± 7.70% and 10.41 ± 6.66% at 512 μg/mL and 256 μg/mL, respectively. The promotional activity of Fr.3 toward *Aspergillus* sp. CD-3 was very obvious at 16–512 μg/mL (Figure 3H). At 256 μg/mL, the promotion rates reached 57.56 ± 14.51% at 72 h.

These results suggested that the active substances in *V. negundo* are present in Fr.3, and that these compounds are able to promote the formation of broad bean koji. Therefore, Fr.3 was subjected to further and more detailed study.

### 3.7 Two prominent chemical substances from *V. negundo* inhibit certain bacterial strains and promote fungal spore germination

Chromatographic separation and nuclear magnetic resonance (NMR) spectroscopy analyses were conducted (Supplementary Figures 9–16), and the compounds HJ-1 and HJ-2 were isolated from Fr.3 of the petroleum ether extract of *V. negundo* leaves. To confirm the function of two compounds in the manufacture of broad bean koji, these two compounds were tested for biological activity toward the six study strains of bacteria (*S. aureus*, *B. subtilis*, *B. wiedmannii* cdxj-1, *P. putida* cdxj-2, *B. albus* cdxj-3, *B. aerius* cdxj-4) and two strains of fungi (*Rhizopus* sp. CD-1, and *Aspergillus* sp. CD-3).

**FIGURE 4:**
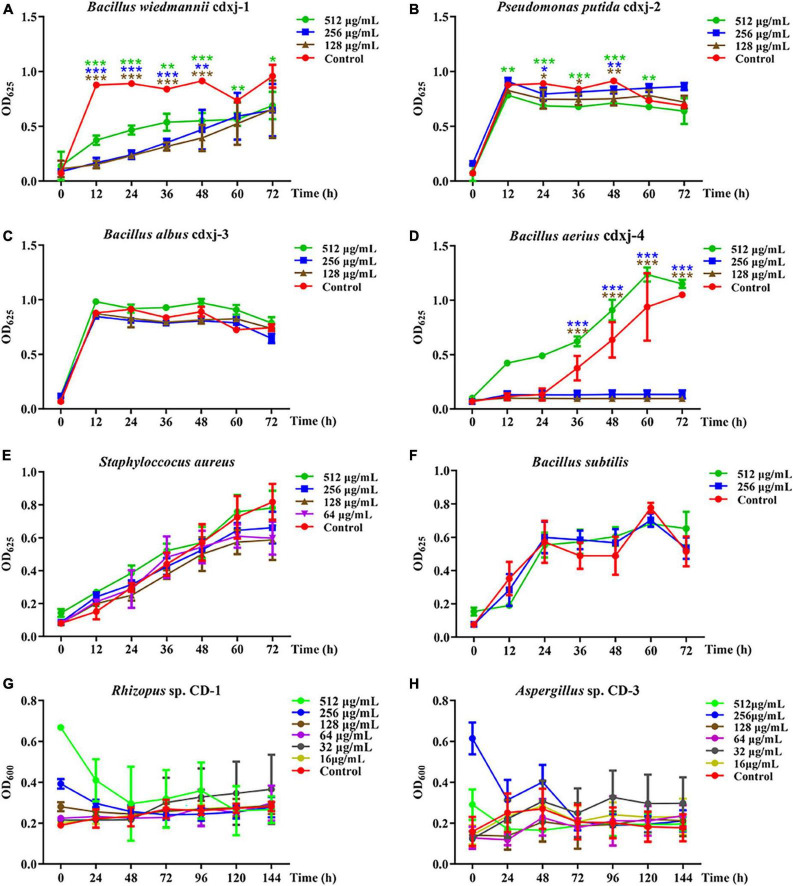
The biological activity of compound HJ-1 from *Vitex negundo* toward bacteria and fungi. **(A–F)** Compound HJ-1 inhibits the growth of certain strains of bacteria, including *B. wiedmannii* cdxj-1 **(A)**, *P. putida* cdxj-2 **(B)**, *B. albus* cdxj-3 **(C)**, *B. aerius* cdxj-4 **(D)**, *S. aureus* **(E)**, and *B. subtilis* **(F)**. **(G,H)** Compound HJ-1 promotes the growth of certain fungi, including *Rhizopus* sp. CD-1 **(G)** and *Aspergillus* sp. CD-3 **(H)**. Triple asterisks indicate significant differences between the control group and other treatments, ***p < 0.001, **p < 0.05, *p < 0.01.

HJ-1 had antibacterial activities against *B. wiedmannii* cdxj-1 at concentrations of 128 μg/mL concentrations at 48 h, and displayed remarkable inhibitory activity with a suppression rate of 27.99 ± 13.10% at 512 μg/mL, at 72 h (Figure 4A). Moreover, HJ-1 was also able to inhibit the growth of *P. putida* cdxj-2, particularly at concentrations of 512 μg/mL. After 48 h, the inhibition rates of HJ-1 at concentrations of 512 μg/mL, 256 μg/mL and 128 μg/mL against *P. putida* cdxj-2 were 22.09 ± 1.54%, 9.11 ± 3.53% and 17.79 ± 6.74%, respectively (Figure 4B). HJ-1 exhibited significant growth inhibition activity against *B. aerius* cdxj-4 between 36–72 h, and the inhibition rate of HJ-1 at 128 μg/mL toward *Bacillus* sp. cdxj-4 was 90.84 ± 1.55% at 72 h (Figure 4D). Nevertheless, HJ-1 had no obvious inhibitory activity against the bacterial strains *B. albus* cdxj-3, *S. aureus* or *B. subtilis* (Figures 4C, E, F).

HJ-1 had no inhibitory effect on either of the two fungal strains isolated from broad bean koji, *Rhizopus* sp. CD-1 and *Aspergillus* sp. CD-3 (Figures 4G, H). However, after 48 h, *Aspergillus* sp. CD-3 spores could be observed germinating in the group co-cultured with HJ-1, while no spores germinated in the control group (Supplementary Figure 8A). The OD values did not change significantly between the control and HJ-1 co-culture group. Therefore, HJ-1 is thought to promote the conidial germination of *Aspergillus* sp. CD-3.

**FIGURE 5:**
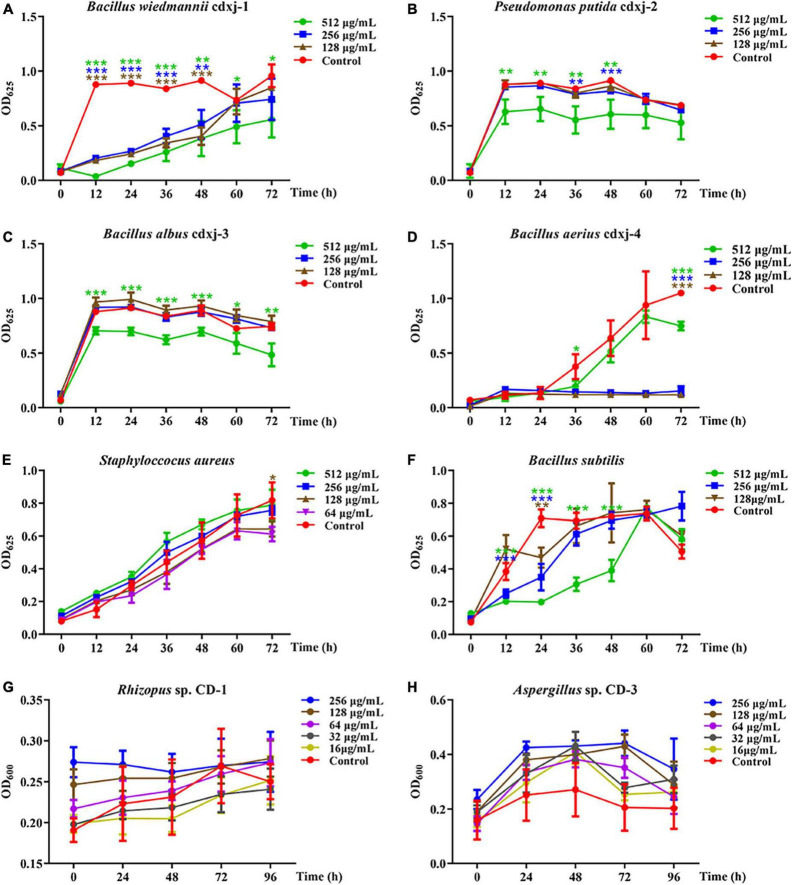
The biological activity of compound HJ-2 from *Vitex negundo* toward bacteria and fungi. **(A–F)** Compound HJ-2 inhibits the growth of certain strains of bacteria, including *B. wiedmannii* cdxj-1 **(A)**, *P. putida* cdxj-2 **(B)**, *B. albus* cdxj-3 **(C)**, *B. aerius* cdxj-4 **(D)**, *S. aureus* **(E)**, and *B. subtilis* **(F)**. **(G,H)** Compound HJ-2 promotes the growth of certain fungi, including *Rhizopus* sp. CD-1 **(G)** and *Aspergillus* sp. CD-3 **(H)**. Triple asterisks indicate significant differences between the control group and other treatments, ***p < 0.001, **p < 0.05, *p < 0.01.

HJ-2 had obvious inhibitory activity against *B. wiedmannii* cdxj-1 (Figure 5A). At 48 h, 128 μg/mL of HJ-2 had an inhibition rate of 55.73 ± 8.77%, and an IC_50_ value of 209.09 ± 76.17 μg/mL. The growth inhibition activity of HJ-2 was weak toward *P. putida* cdxj-2, with inhibition rates of only 33.83 ± 14.67% following co-culture with 512 μg/mL HJ-2 at 48 h (Figure 5B). Similarly, the growth of *B. albus* cdxj-3 was inhibited only weakly by HJ-2 in high concentrations (512 μg/mL), with an inhibitory rate of 35.07 ± 13.99% at 72 h (Figure 5C). Interestingly, HJ-2 showed strong growth inhibition activity against *B. aerius* cdxj-4 (Figure 5D). After treatment for 36 h, 128 μg/mL of HJ-2 began to inhibit *B. aerius* cdxj-4, and reached the most powerful inhibitory effect at 72 h, with an inhibition rate of 88.86 ± 0.66% and an IC_50_ value of 394.88 ± 14.52 μg/mL. HJ-2 displayed little growth inhibitory activity against *S. aureus* (Figure 5E). Moreover, HJ-2 also exhibited a remarkable inhibitory effect against *B. subtilis* (Figure 5F). Within 48 h of treatment, HJ-2 at concentrations of 256 μg/mL and 512 μg/mL both effectively delayed the logarithmic growth phase of *B. subtilis*. The inhibitory activity of HJ-2 was most obvious at 24 h, with inhibition rates of 72.02 ± 2.02%, 50.72 ± 11.47%, and 33.73 ± 8.55% at 512 μg/mL, 256 μg/mL, and 128 μg/mL, respectively.

HJ-2 exhibited no inhibitory activity toward the growth of the fungi *Rhizopus* sp. CD-1 and *Aspergillus* sp. CD-3 (Figures 5G, H). Under an optical microscope, germinated spores of *Aspergillus* sp. CD-3 were observed following co-culture for 48 h with HJ-2 (Supplementary Figures 8B, C), in contrast to the control, where very few spores germinated. The OD value of the treatments did not change significantly. Thus, HJ-2 is able to promote the germination of *Aspergillus* sp. CD-3 spores.

These results demonstrated that the two polymethoxylated flavones HJ-1 and HJ-2 are active components of *V. negundo* leaves, and can inhibit the growth of certain bacterial strains and promote the growth of certain fungi during the formation of broad bean koji.

### 3.8 Quantification of HJ-1 and HJ-2 in fresh *V. negundo* leaves

**FIGURE 6:**
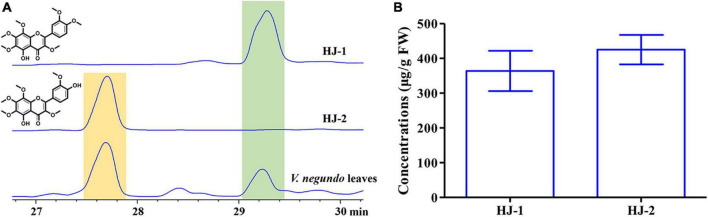
Qualitative and quantitative analyses of compounds HJ-1 and HJ-2 isolated from the fresh leaves of *V. negundo*. **(A)** Qualitative analyses of compounds HJ-1 and HJ-2 using HPLC at 210 nm. **(B)** Quantitative analyses of compounds HJ-1 and HJ-2.

The concentrations of HJ-1 and HJ-2 in fresh *V. negundo* leaves were determined using a quantitative HPLC analysis (Figure 6A). The concentration of HJ-1 in fresh *V. negundo* leaves was found to be 363.95 ± 57.74 μg/g FW, while the concentration of HJ-2 in fresh *V. negundo* leaves was 425.09 ± 42.58 μg/g FW (Figure 6B).

## 4 Discussion

### 4.1 *V. negundo* leaves are able to promote the fermentation process during the manufacture of broad bean koji

Broad bean paste is a popular traditional fermented food in Asia and worldwide, and has excellent nutritional value (Zhao et al., 2021; Yu et al., 2022). However, the traditional fermentation method to make broad bean koji is extremely long and difficult (Li et al., 2019). *V. negundo* Linn. (Verbenaceae) is widely used to make broad bean koji in Sichuan, China, but the efficacy of *V. negundo* in this process has not been substantiated until now, and the responsible compounds have not to date been identified. In this study, we investigated the manufacture of broad bean koji using *V. negundo* leaves. We found that covering the fermenting beans with *V. negundo* leaves effectively promoted the fermentation process, which was observably beneficial for the generation of koji. Moreover, the success rate was higher and the probability of decay and deterioration was reduced compared with control. These results provide a scientific basis for the application of *V. negundo* leaves in the production of broad bean koji.

### 4.2 Microorganisms play an essential role in the manufacture of broad bean koji

Broad bean koji is formed through the natural fermentation of various microorganisms (Zhao et al., 2022). The techniques of broad bean koji manufacture differ between regions. However, microorganisms always play significant metabolic roles in the process, and can have a significant impact on the quality of the resulting paste. The formation of color, shape of high-quality, flavor and the rate of deterioration are dependent on these organisms, with *Aspergillus oryzae* being of particular importance (Liu et al., 2020; Lin et al., 2021; Yang Y. et al., 2021). *A. oryzae* is typically the dominant strain in the koji-making process, although certain other fungal taxa have also been found, including *Saccharomycetales*, *Issatchenkia*, *Cladosporium*, *Rhizopus* sp., and *Sterigmatomyces* (Feng et al., 2007; Yang M. L. et al., 2021). In this study, we isolated two dominant fungi from the mature koji generated using *V. negundo* leaves during the fermentation. The fungi were identified as *Rhizopus* sp. CD-1 and *Aspergillus* sp. CD-3, which is consistent with the results of previous research, and indicates that *V. negundo* leaves can promote the fermentation of the koji but do not change the dominant species or dominant strains involved in the process. Moreover, four dominant strains of bacteria were obtained from failed koji, and were identified as *Bacillus wiedmannii* cdxj-1, *Pseudomonas putida* cdxj-2, *B. albus* cdxj-3, and *B. aerius* cdxj-4. These strains have also been found in food or rotting food in past studies. Especially *P. putida* cdxj-2, *P. putida* was discovered being the predominant spoilage organisms in many spoiled foods, such as it was dominant bacteria of spoiled chicken breast meat in previous studies (Zhao et al., 2019; Duan et al., 2020; Kumar et al., 2023). Hence, it is significant to promote these fungal growth and inhibit these bacterial growth to improve the effect on making koji of broad bean paste.

### 4.3 Polymethoxylated flavones are responsible for the effects of *V. negundo* on koji-making microorganisms

Secondary metabolites are an important medium for plants to interact with the outside world and exercise their functions (Bennett and Wallsgrove, 1994; Piasecka et al., 2015; Li et al., 2017). They have a wide range of biological activities (Vaishnav and Demainb, 2010; Liu et al., 2023), and some metabolites are used in functional foods or dietary supplements by virtue of their effects on the growth and fermentation of microorganisms, to serve as natural preservatives, or to prevent foods from oxidative deterioration (Sanchez Maldonado et al., 2015). In this study, we investigated secondary metabolites in *V. negundo* leaves based on their biological activities. The chemical substances present in *V. negundo* leaves were extracted using petroleum ether or methanol extractions, and were then co-cultured with the isolated fungi and bacteria. We found that these extractions from *V. negundo* leaves demonstrated inhibitory activity against the growth of bacteria, and could also promote the growth of the target fungi. And petroleum ether extract of *V. negundo* leaves displayed more significant activities toward microorganisms, and inhibition rate could reach 90% against bacteria at 512 μg/mL and 256 μg/mL. In addition, their petroleum ether extract had good promotion rates to fungi *Rhizopus* sp. CD-1 and *Aspergillus* sp. CD-3. Hence, petroleum ether extract was subjected to isolation according to activity, and two compounds HJ-1 and HJ-2 were obtained.

Two polymethoxylated flavones, HJ-1 and HJ-2 were found to be the compounds responsible for this bioactivity, and were identified using NMR. Previous studies have identified other flavonoids with observable antibacterial activity (Tim Cushnie and Lamb, 2011; Farhadi et al., 2019), including alpinumisoflavone and burttinone from *Erythrina caffra* Thunb., which have significant inhibitory activity against both Gram-positive and Gram-negative bacteria (Chukwujekwu et al., 2011). In addition, some flavonoids have been shown to promote fungal spore germination, for example, the flavonoids quercetin and rutin, which are able to promote spore germination in *Rhizophagus irregularis* (Lidoy et al., 2022).

HJ-1 and HJ-2 both showed good inhibitory activity against certain bacterial strains, and both were able to promote spore germination in *Rhizopus* sp. CD-1 and *Aspergillus* sp. CD-3. The concentrations of HJ-1 and HJ-2 in fresh *V. negundo* leaves were found to be 363.95 ± 57.74 and 425.09 ± 42.58 μg/g, respectively, which was well within their effective concentrations when interacting with the tested microorganisms. These results indicated that the two polymethoxylated flavones HJ-1 and HJ-2 were the active components in *V. negundo* leaves and that these compounds were able to effectively improve the manufacturing process of broad bean koji. We believe that this work will provide the basis for a large number of applications of *V. negundo* leaves in the production of broad bean paste.

## 5 Conclusion

Two dominant fungal strains were obtained from mature broad bean koji made using *V. negundo* leaves, *Rhizopus* sp. CD-1 and *Aspergillus* sp. CD-3. Four dominant bacterial strains were obtained from rotten beans resulting from failed koji, *B. wiedmannii* cdxj-1, *P. putida* cdxj-2, *B. albus* cdxj-3, and *B. aerius* cdxj-4. The effects of secondary metabolites of *V. negundo* on the growth of two fungi and six bacteria were investigated, and the petroleum ether extract of *V. negundo* was found to effectively promote fungal growth and inhibiting that of the bacteria. Two flavonoid compounds, HJ-1 and HJ-2, were isolated from *V. negundo* leaves using column chromatography, spectral identification and activity assays. HJ-1 and HJ-2 were able to inhibit the growth of several strains of bacteria and promote the spore germination of the two isolated fungal strains. HJ-1 and HJ-2 are therefore considered to be the active substances in *V. negundo* responsible for the observed promotion of the broad bean koji fermentation process. This study revealed the active compounds in *V. negundo* leaves and their effects toward various microorganisms important in the broad bean koji process, and established a scientific basis for the use of *V. negundo* in broad bean koji production.

## Data availability statement

The datasets presented in this study can be found in online repositories. The names of the repository/repositories and accession number(s) can be found in this article/Supplementary material.

## Author contributions

JL: Conceptualization, Formal analysis, Investigation, Methodology, Software, Supervision, Validation, Visualization, Writing – original draft, Writing – review & editing. YX: Data curation, Formal analysis, Investigation, Writing – original draft. JY: Conceptualization, Data curation, Resources, Writing – original draft. LB: Data curation, Formal analysis, Investigation, Software, Writing – original draft. JH: Investigation, Methodology, Project administration, Resources, Software, Validation, Writing – original draft, Writing – review & editing. SL: Conceptualization, Data curation, Formal analysis, Funding acquisition, Investigation, Methodology, Project administration, Resources, Supervision, Validation, Writing – original draft, Writing – review & editing.
