# Supplementary material for: Polymethoxylated flavones from the leaves of Vitex negundo have fungal-promoting and antibacterial activities during the production of broad bean koji
Source: Front Microbiol. 2024 May 1;15:1401436. doi: 10.3389/fmicb.2024.1401436 (PMC11094617; doi:10.3389/fmicb.2024.1401436)
Supplement: Supplementary file 1 [file Table_1.docx]

Polymethoxylated flavones from the leaves of Vitex negundo have fungal-promoting and antibacterial activities during the production of broad bean koji

Supplementary Material

Jiayi Liu, Yetong Xu, Jianyu Yan, Liping Bai, Juan Hua*, Shihong Luo*

Research Center of Protection and Utilization of Plant Resources, College of Bioscience and Biotechnology, Shenyang Agricultural University, Shenyang, 110866, Liaoning Province, China

^2^Laboratory X, Institute X, Department X, Organization X, City X, State XX (only USA, Canada and Australia), Country

*** Correspondence:**

Juan Hua

huajuan@syau.edu.cn;

Shihong Luo

luoshihong@syau.edu.cn (http://orcid.org/0000-0003-3500-3466)

**List of Supplementary Information:**

**Figure S1.** Phylogenetic tree of *Rhizopus* sp. CD-1 based on 15 DNA barcode sequences.

**Figure S2.** Phylogenetic tree of *Aspergillus* sp. CD-3 based on 15 DNA barcode sequences.

**Figure S3.** Phylogenetic tree of *Bacillus Wiedmannii* cdxj-1 based on 15 DNA barcode sequences.

**Figure S4.** Phylogenetic tree of *Pseudomonas putida* cdxj-2 based on 15 DNA barcode sequences.

**Figure S5.** Phylogenetic tree of *Bacillus albus* cdxj-3 based on 15 DNA barcode sequences

**Figure S6.** Phylogenetic tree of *Bacillus aerius* cdxj-4 based on 15 DNA barcode sequences.

**Figure S7.** The biological activity of the methanol extract of *V. negundo* leaves towards various bacteria.

**Figure S8. HJ-1** and **HJ-2** can promote the spore germination of *Aspergillus* sp. CD-3.

**Figure S9.** ^13^C NMR and DEPT spectra of **HJ-1** recorded at 150 MHz in methanol-*d*_4_.

**Figure S10.** ^1^H NMR spectrum of **HJ-1** recorded at 600 MHz in methanol-*d*_4_.

**Figure S11.** HSQC spectrum of **HJ-1** recorded in methanol-*d*_4_.

**Figure S12.** HMBC spectrum of **HJ-1** recorded in methanol-*d*_4_.

**Figure S13.** ^1^H-^1^H COSY spectrum of **HJ-1** recorded in methanol-*d*_4_.

**Figure S14.** ROESY spectrum of **HJ-1** recorded in methanol-*d*_4_.

**Figure S15.** ^13^C NMR and DEPT spectra of **HJ-2** recorded at 150 MHz in chloroform-*d*.

**Figure S16.** ^1^H NMR spectrum of **HJ-2** recorded at 600 MHz in chloroform-*d*.


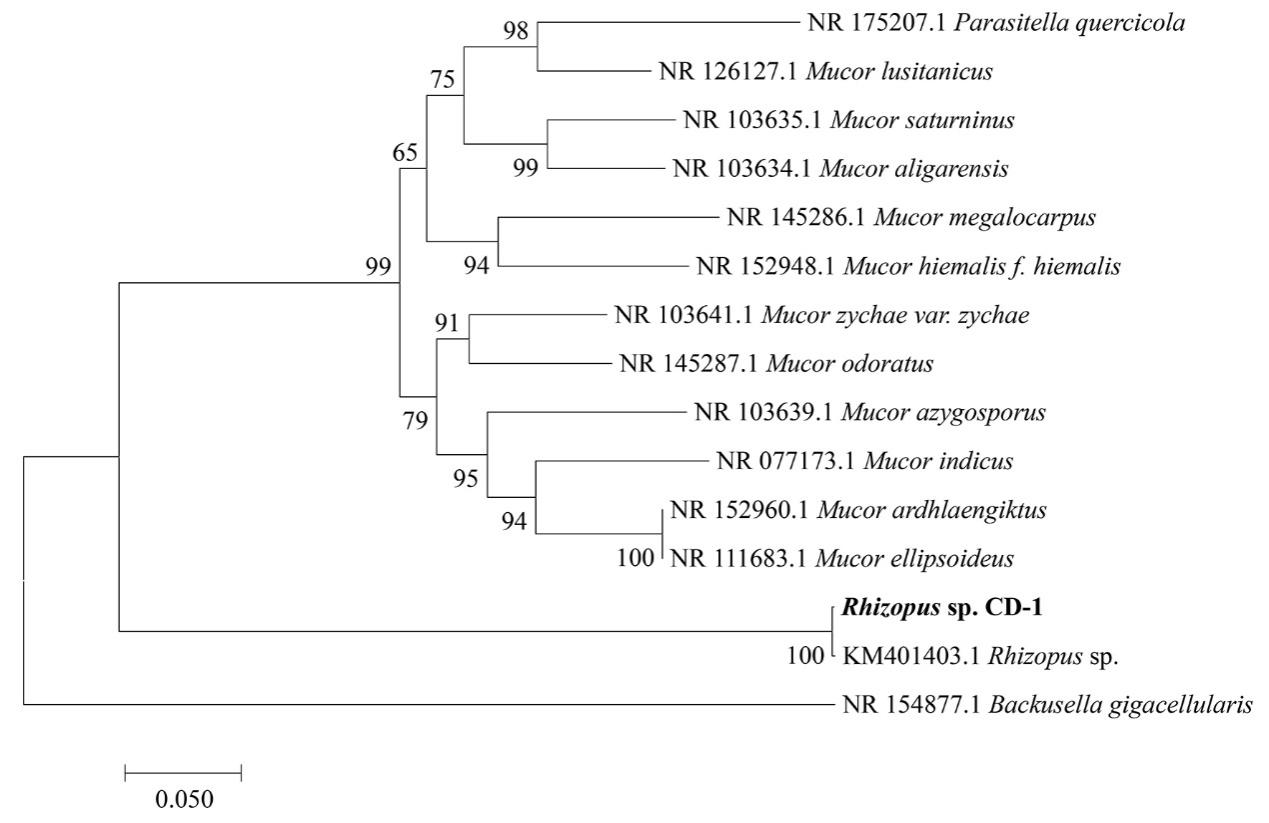


**Figure S1.** Phylogenetic tree of *Rhizopus* sp. CD-1 based on 15 DNA barcode sequences. The tree was reconstructed based on neighbor-joining and was tested using bootstrapping with 1000 replications.


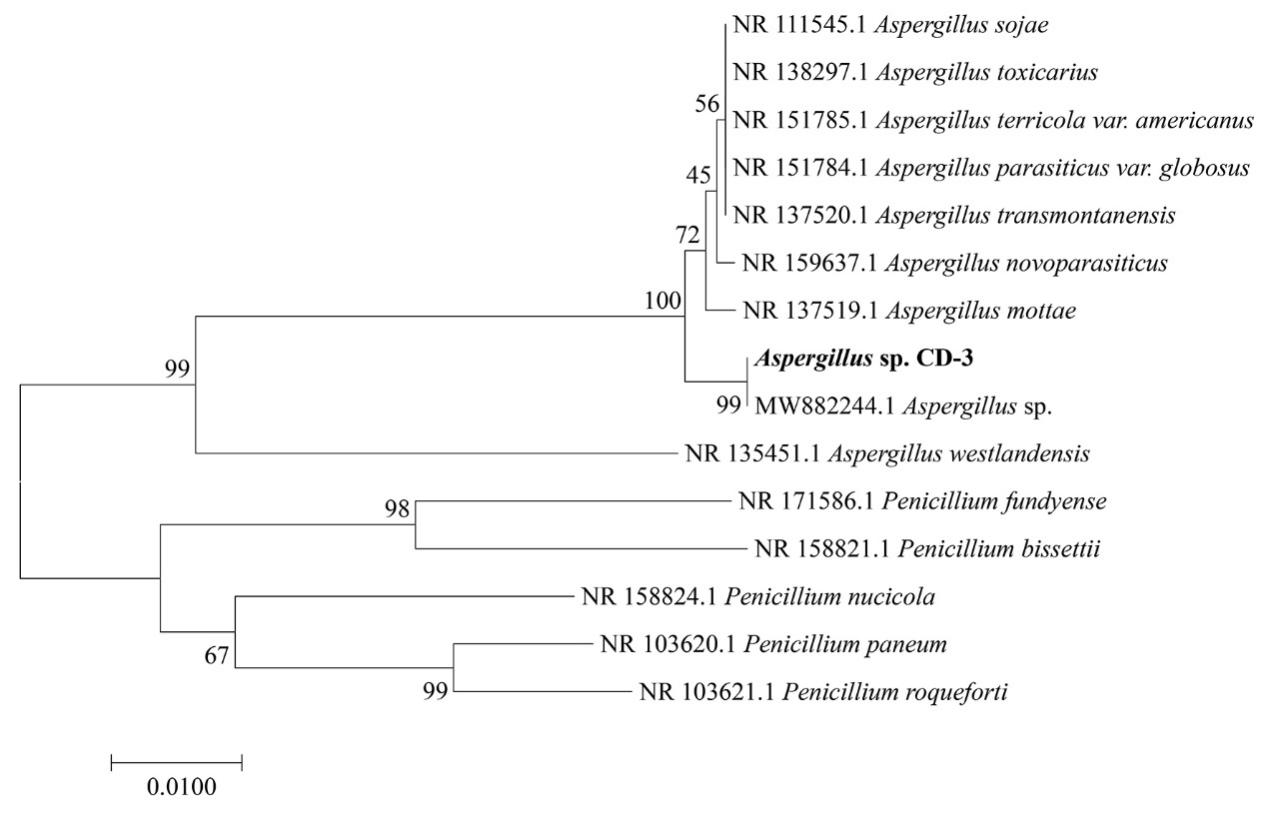


**Figure S2.** Phylogenetic tree of *Aspergillus* sp. CD-3 based on 15 DNA barcode sequences. The tree was reconstructed based on neighbor-joining and was tested using bootstrapping with 1000 replications.


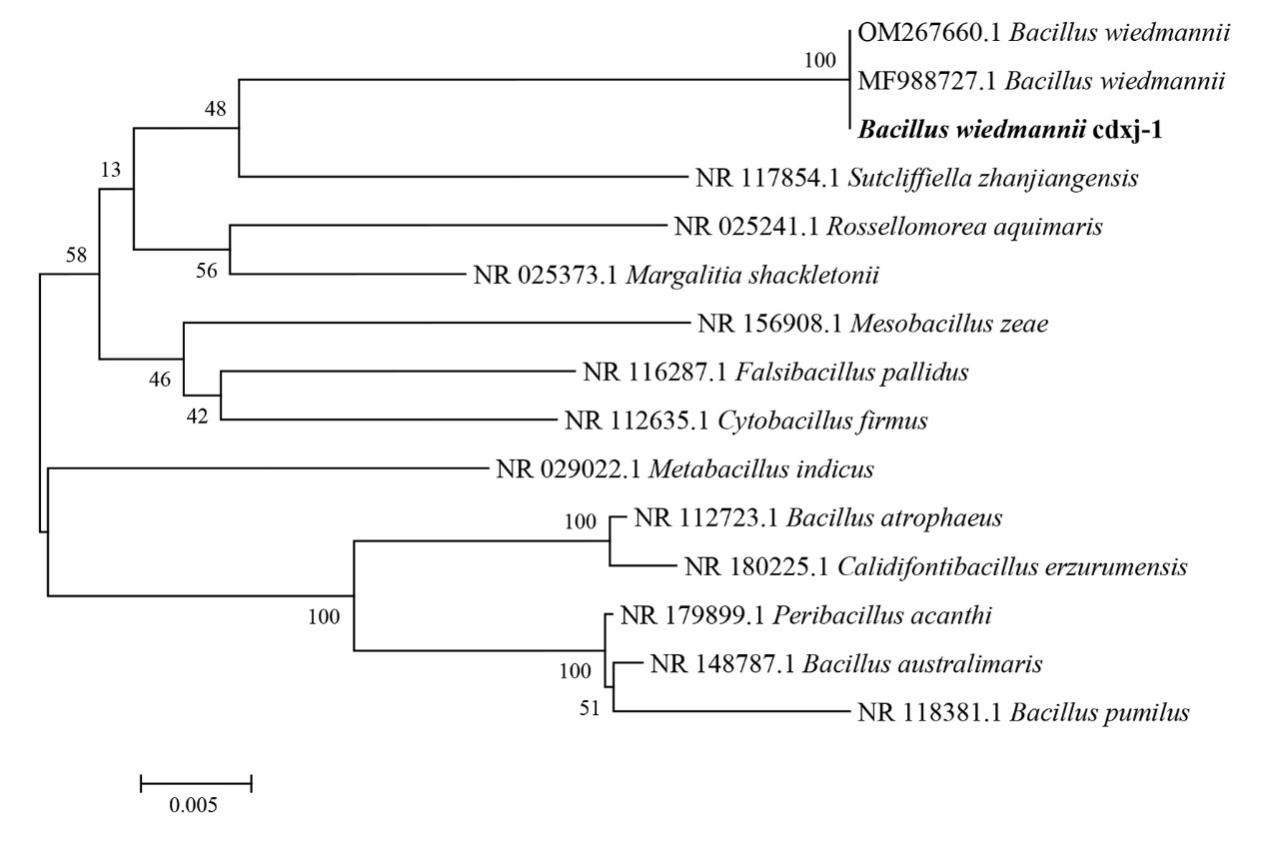


**Figure S3.** Phylogenetic tree of *Bacillus Wiedmannii* cdxj-1 based on 15 DNA barcode sequences. The tree was reconstructed based on neighbor-joining and was tested using bootstrapping with 1000 replications.


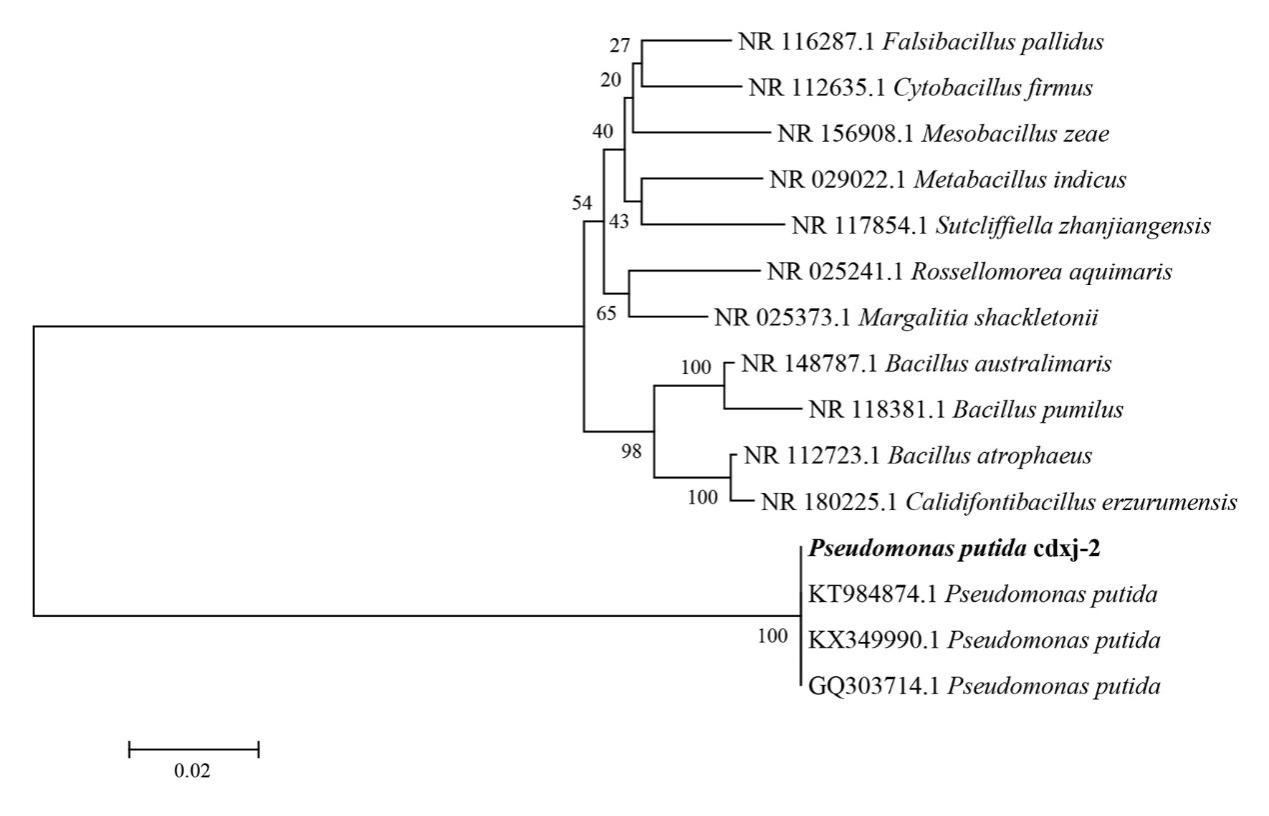


**Figure S4.** Phylogenetic tree of *Pseudomonas putida* cdxj-2 based on 15 DNA barcode sequences. The tree was reconstructed based on neighbor-joining and was tested using bootstrapping with 1000 replications.


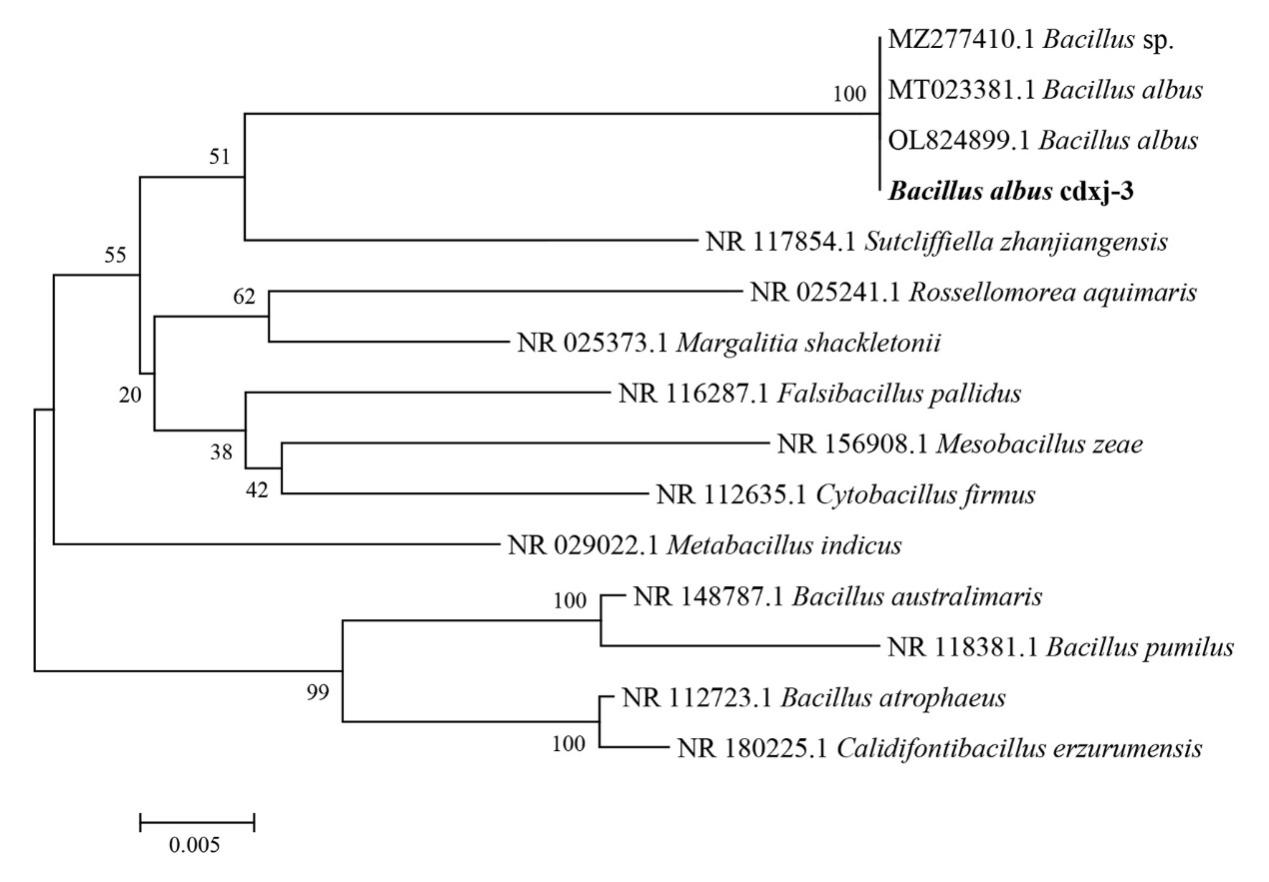


**Figure S5.** Phylogenetic tree of *Bacillus albus* cdxj-3 based on 15 DNA barcode sequences. The tree was reconstructed based on neighbor-joining and was tested using bootstrapping with 1000 replications.


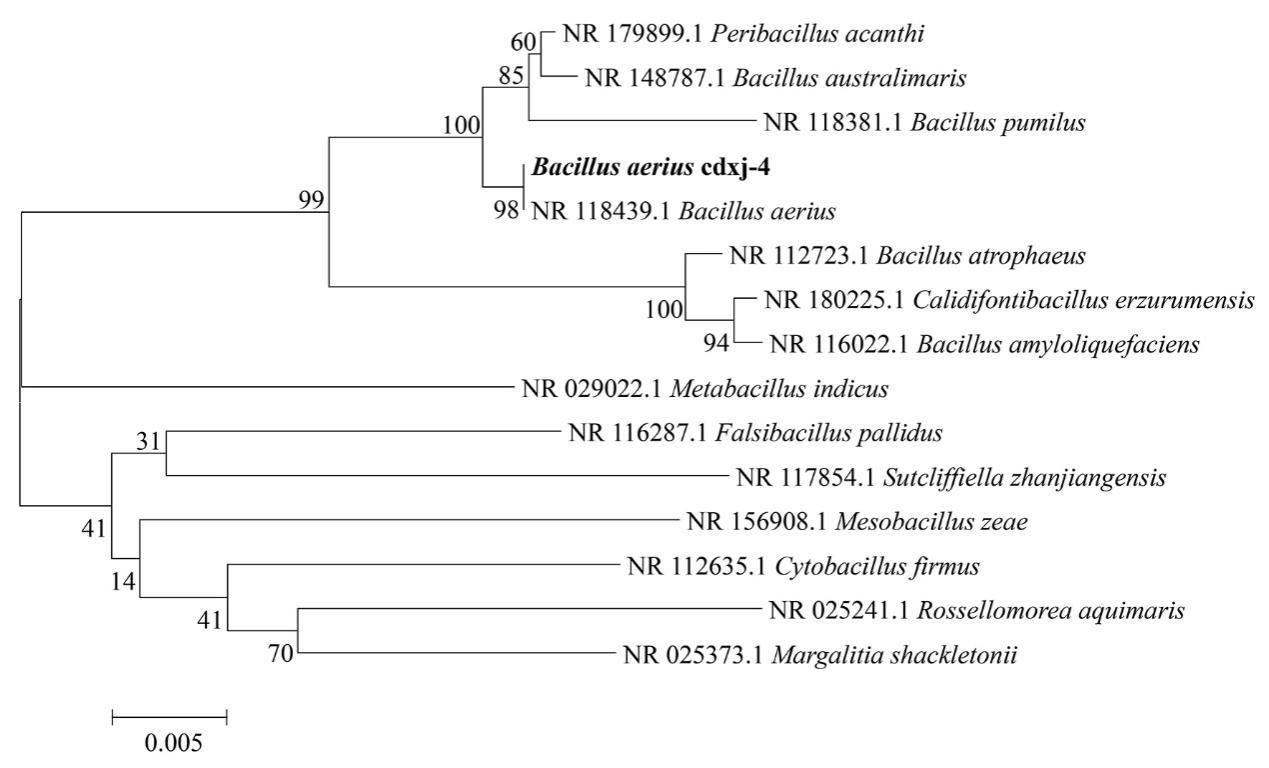


**Figure S6.** Phylogenetic tree of *Bacillus aerius* cdxj-4 based on 15 DNA barcode sequences. The tree was reconstructed based on neighbor-joining and was tested using bootstrapping with 1000 replications.


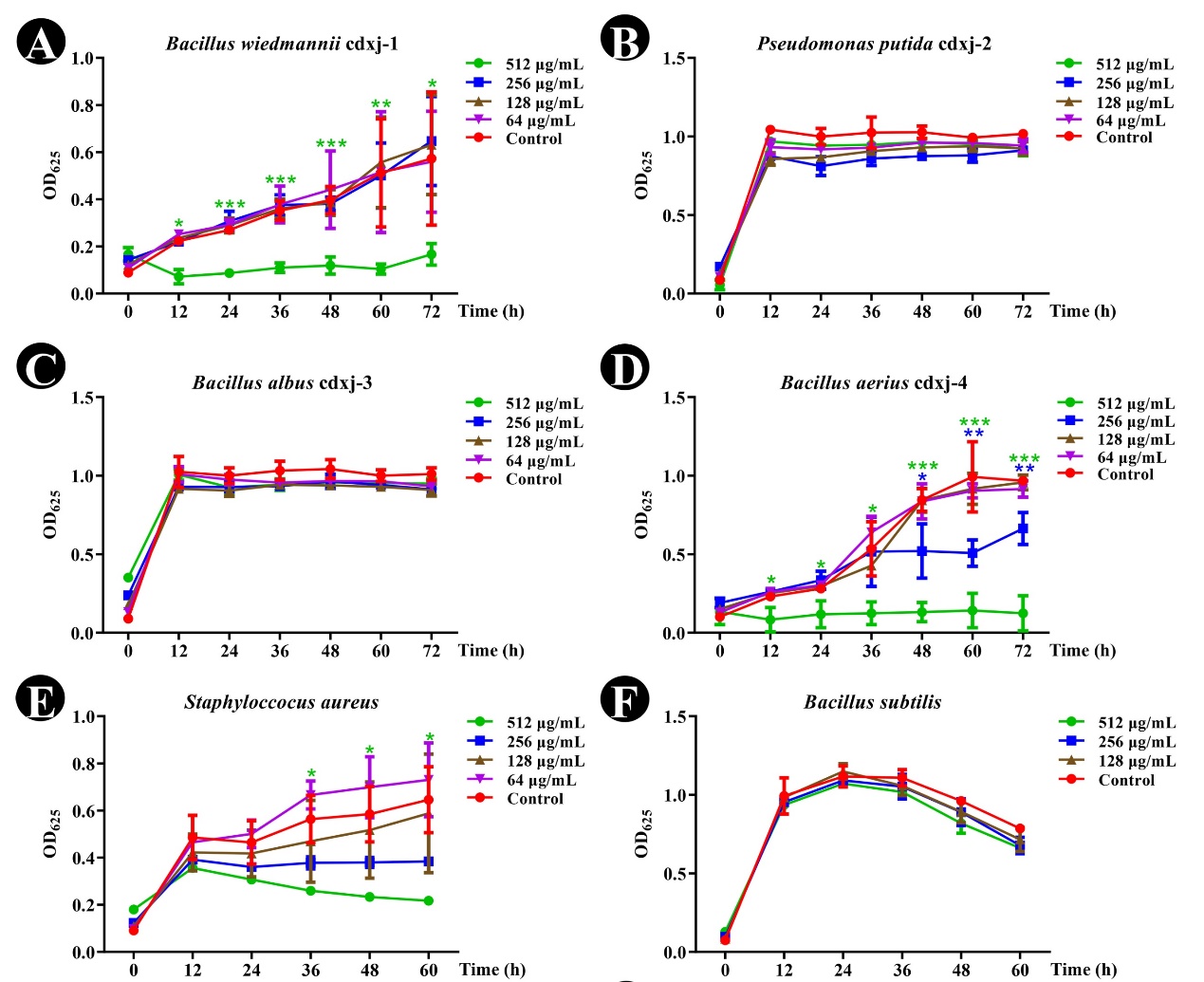


**Figure S7.** The biological activity of methanol extract of *V. negundo* leaves towards bacteria, including *B. wiedmannii* cdxj-1 (A), *P. putida* cdxj-2 (B), *B. albus* cdxj-3 (C), *B. aerius* cdxj-4 (D), *S. aureus* (E), and *B. subtilis* (F).


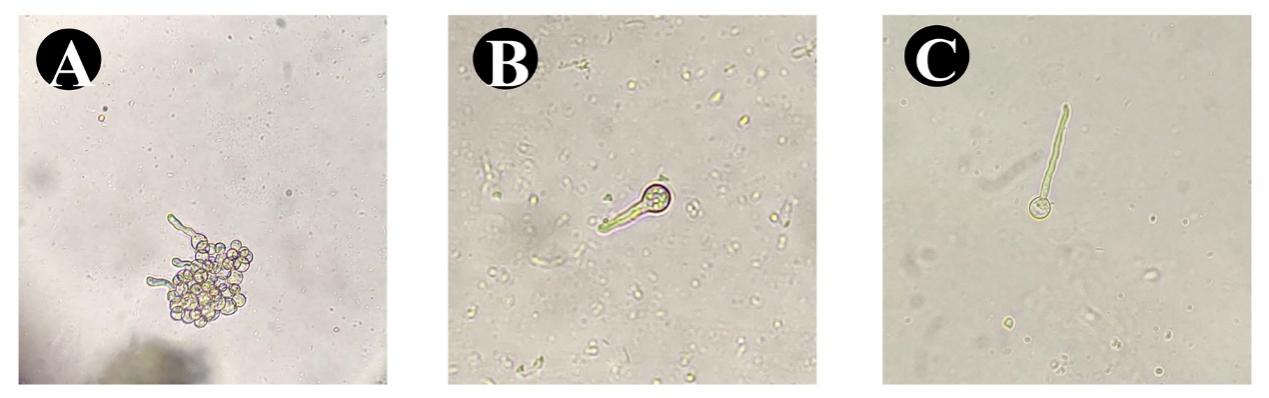


**Figure S8. HJ-1** and **HJ-2** can promote the spore germination of *Aspergillus* sp. CD-3. The spore germination of *Aspergillus* sp.CD-3 cultured for 48 h with **HJ-1** (A) and **HJ-2** (B, C), visualized under a light microscope.

**
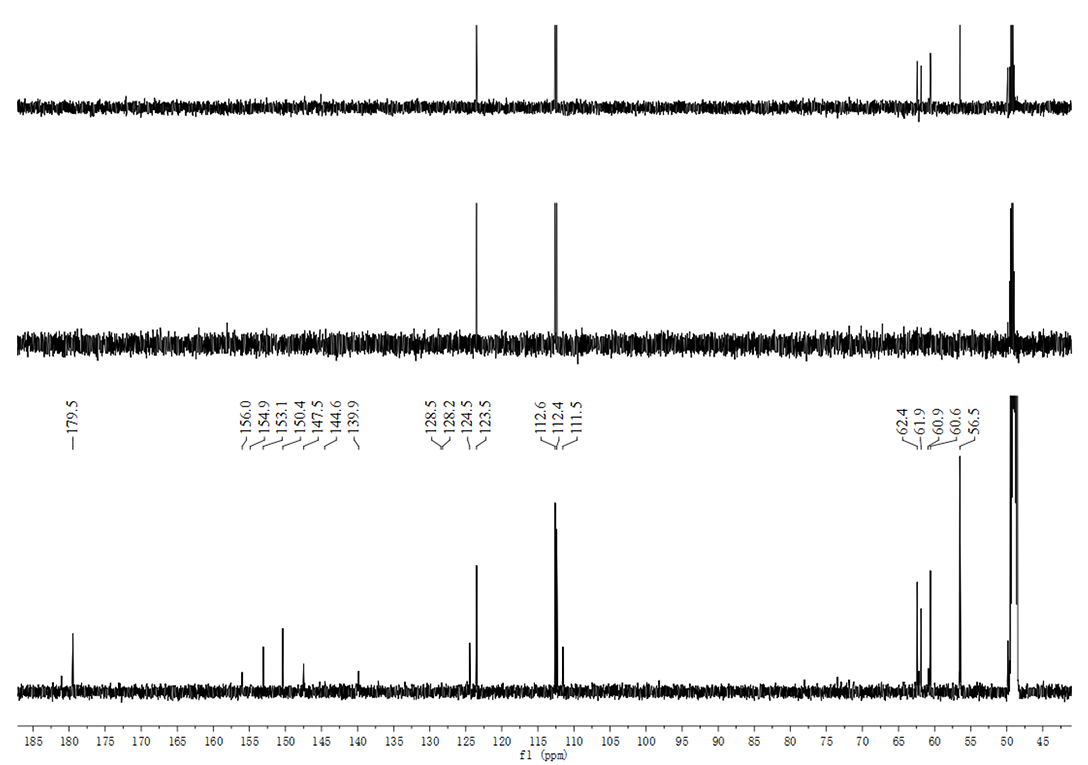
**

**Figure S9.** ^13^C NMR and DEPT spectra of **HJ-1** recorded at 150 MHz in methanol-*d*_4_.

**
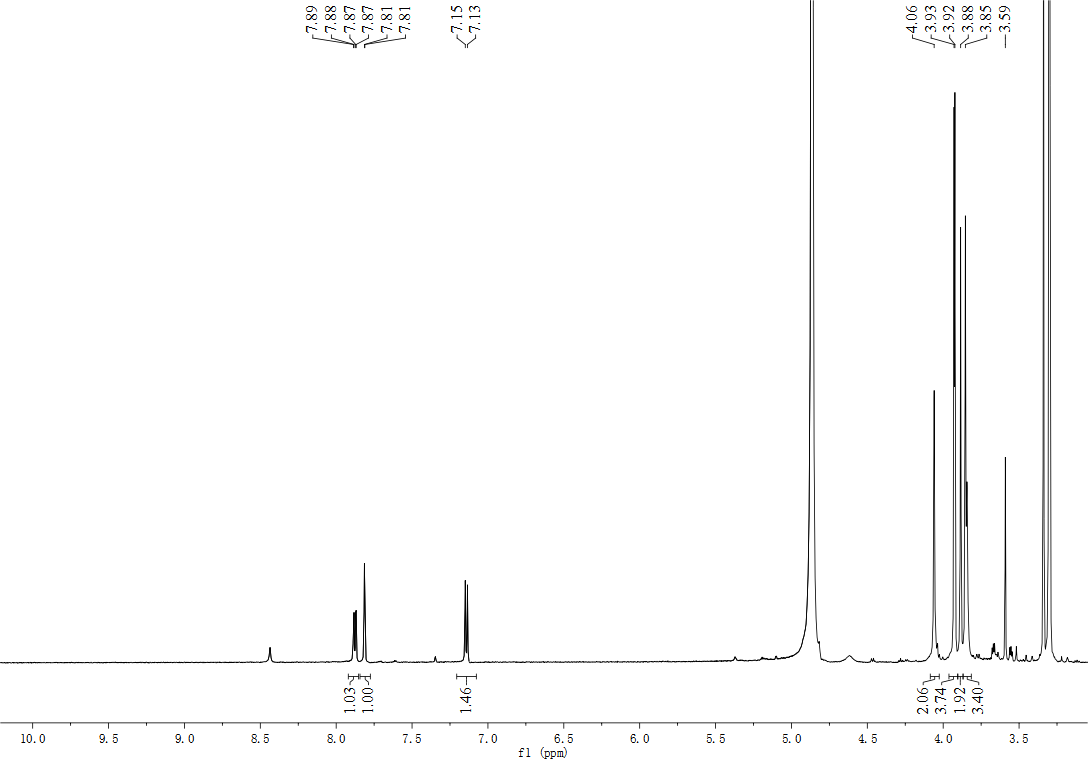
**

**Figure S10.** ^1^H NMR spectrum of **HJ-1** recorded at 600 MHz in methanol-*d*_4_.

**
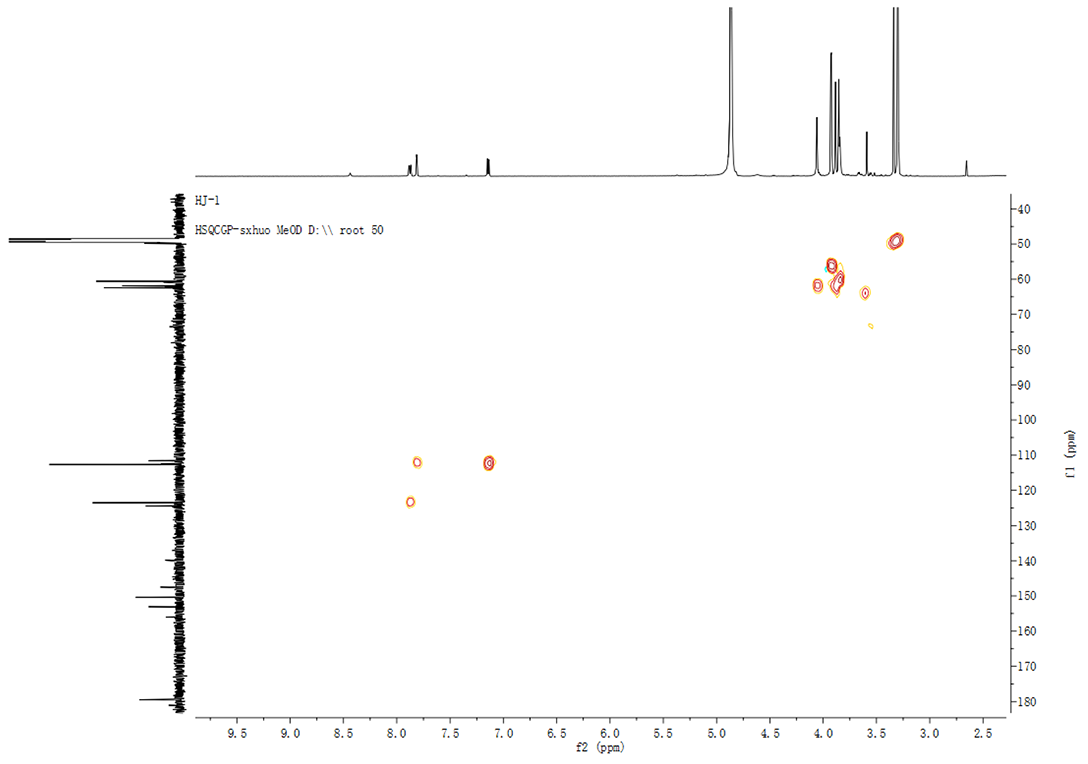
**

**Figure S11.** HSQC spectrum of **HJ-1** recorded in methanol-*d*_4_.

**
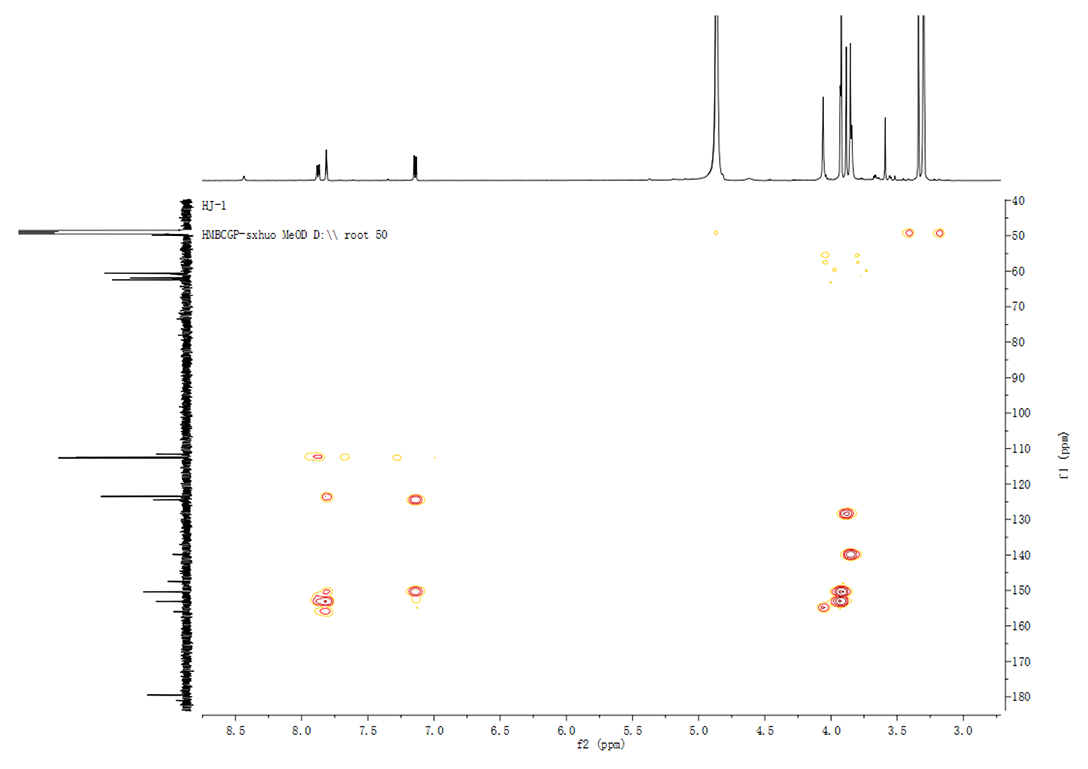
**

**Figure S12.** HMBC spectrum of **HJ-1** recorded in methanol-*d*_4_.

**
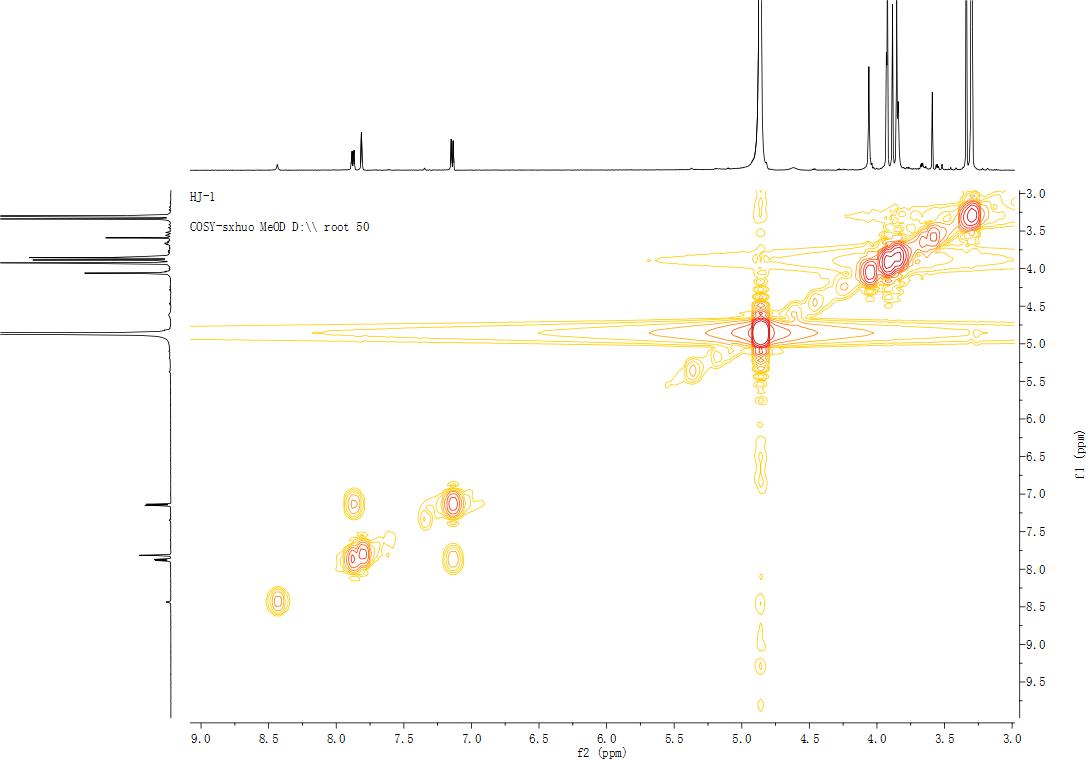
**

**Figure S13.** ^1^H-^1^H COSY spectrum of **HJ-1** recorded in methanol-*d*_4_.


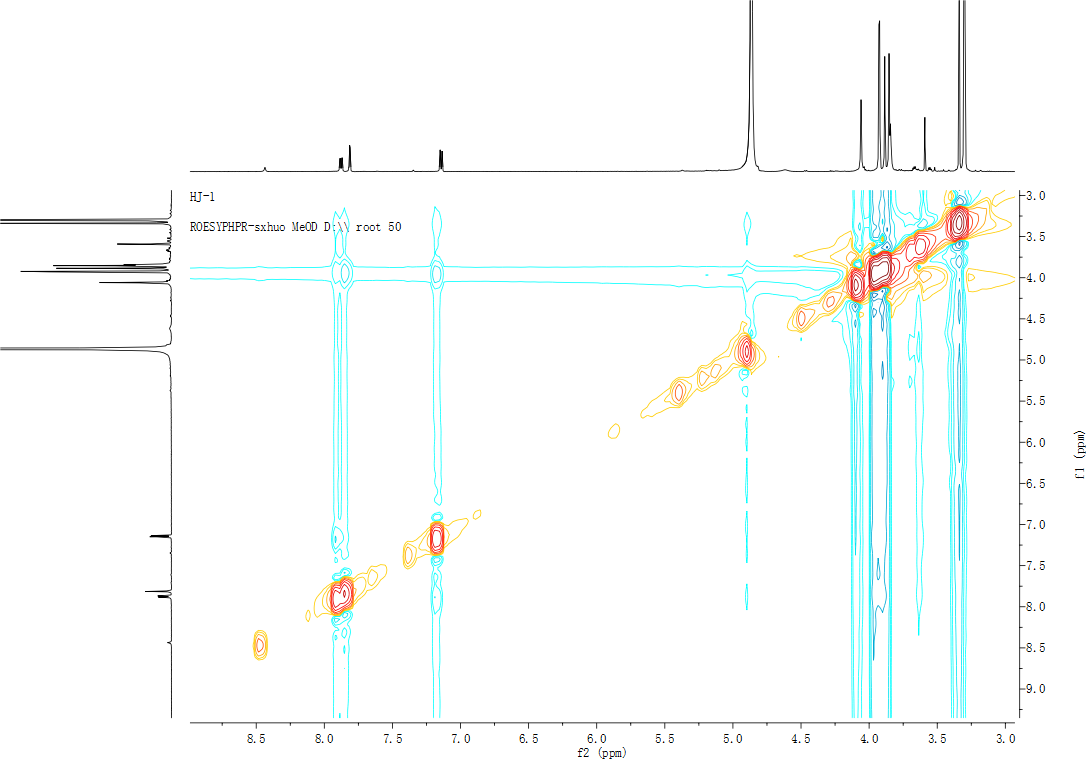


**Figure S14.** ROESY spectrum of **HJ-1** recorded in methanol-*d*_4_.


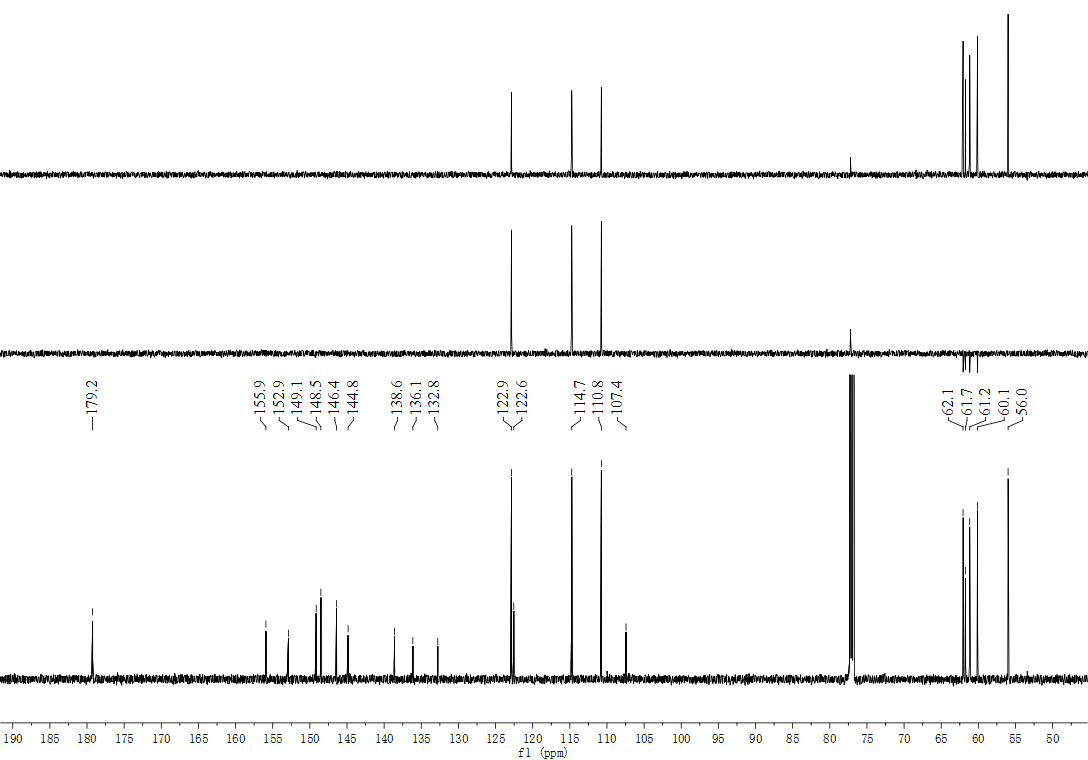


**Figure S15.** ^13^C NMR and DEPT spectra of **HJ-2** recorded at 150 MHz in chloroform-*d*.


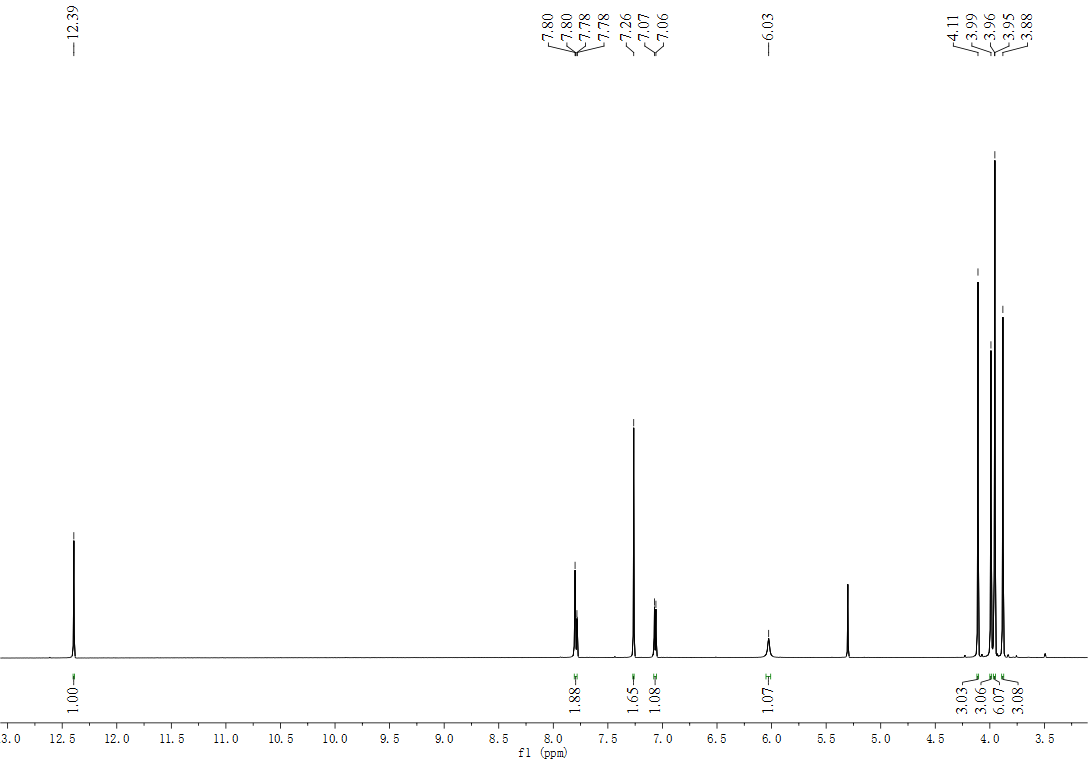


**Figure S16.** ^1^H NMR spectrum of **HJ-2** recorded at 600 MHz in chloroform-*d*.
